# Statistical validity and reliability of the Persian version of the Western Ontario Meniscal Evaluation Tool (WOMET) according to the COSMIN checklist

**DOI:** 10.1186/s12891-020-3171-2

**Published:** 2020-03-23

**Authors:** Naghmeh Ebrahimi, Soofia Naghdi, Noureddin Nakhostin Ansari, Shohreh Jalaie, Nasser Salsabili

**Affiliations:** 1 Department of Physiotherapy, Faculty of Rehabilitation, Tehran University of Medical Sciences, Tehran, Iran; 2 Student Research Committee, School of Rehabilitation Sciences, Shiraz University of Medical Sciences, Shiraz, Iran

**Keywords:** WOMET, Meniscus, Translation, Validity, Reliability, Quality of life, COSMIN

## Abstract

**Background:**

The Western Ontario Meniscal Evaluation Tool (WOMET) is the only questionnaire available to assess quality of life in patients with isolated meniscal injuries. The aims of this study were to prepare the Persian version of the WOMET (PWOMET) and validate it in Iranian patients with isolated meniscal tears.

**Methods:**

In the first stage, the English version of WOMET was translated into Persian. Content validity, and qualitative and quantitative (impact score) face validity were tested by specialists and in a sample of 30 patients. In the second stage, PWOMET was assessed for the evaluation of psychometric properties in 100 patients with isolated meniscal injury and 50 healthy people based on the COSMIN checklist. Construct validity was tested based on structural validity (factor analysis) and hypothesis testing. Correlation with the total scores on the SF-36, IKDC and KOOS were used for concurrent criterion validity. Test-retest reliability and internal consistency were calculated using intraclass correlation coefficient (ICC) and Cronbach’s alpha, respectively. In addition the standard error of measurement (SEM) and smallest detectable change were calculated. Interpretability was investigated as the ceiling and floor effects and minimal important difference.

**Results:**

The PWOMET had acceptable qualitative face validity and content validity. The impact score (quantitative face validity) was more than 1.5 for all items. For construct validity, structural validity (factor analysis) and hypothesis testing ability were confirmed. Correlations between the PWOMET total score and IKDC, SF-36, KOOS scores were 0.61, 0.54 and 0.63, respectively (*p* < 0.001), thus confirming concurrent criterion validity. The intraclass correlation coefficient, Cronbach’s alpha, SEM and smallest detectable change for the PWOMET were 0.73, 0.89, 9.43 and 26.13, respectively. The PWOMET had no ceiling or floor effects, and minimal important difference was 9.07.

**Conclusion:**

The PWOMET provides valid and reliable scores for assessment of the quality of life in patients with isolated meniscal injury.

## Background

One of the most common orthopedic conditions is meniscal injury. Meniscal rupture occurs most often in the third, fourth and fifth decades of human life [1], and the mean incidence of meniscal tear is about 60–70 per 100,000. Meniscal arthroscopy is performed for two milion persons annually worldwide, at a cost of several million USD [2, 3]. People with chronic meniscal injury are at greater risk of increased loads on the knee cartilage – a type of injury that can be associated with knee osteoarthrities. In addition, prolonged meniscal pathology can make people unwell in other ways [2, 4–7]. Previous studies found that neither surgical nor nonsurgical approaches to treat meniscal pathology prevent knee osteoarthritis, although conservative treatments may be more effective in preventing knee osteoarthritis more than partial menisectomy. Because knee osteoarthrities progresses, knee replacement becomes necessary, at considerable cost to the individual and society [3]. Meniscal pathology can cause symptomes such as pain, locking, and swelling, which can affect daily activities, recreational and sport activities, mental states, working conditions and ultimately quality of life.

To determine the efficacy and cost-effectiveness of treatments for orthopedic problems and the effect of symptoms and problems associated with meniscal rupture on the life course of individuals, investigators and clinicians need instruments such as quality of life assessment tools [8, 9]. As a type of patient-centered tool, health-related quality of life assessment tools are used to measure outcomes, and are designed in both general and special formats. The generic type is used in a wide variety of populations and therapeutic interventions, while special tools can be used for specific therapeutic interventionsor demographic groups [10]. Questionnaires are one type of these outcome measures.

The Western Ontario Meniscal Evaluation Tool (WOMET), designed in 2007 by Kirkley and colleagues in the United Kingdom [11], is the first instrument designed to evaluate health-related quality of life in people with meniscal injuries, and measures the symptoms most often associated with meniscal rupture. To date the original version of WOMET, which is in English, has been translated only into Turkish [12], Finnish [13], Chinese [14], German [15] and Dutch [16]. The questionnaire consists of 16 items that cover three areas: 9 questions about Physical symptoms, 4 questions about Sports/recreation/work/lifestyle, and 3 questions about Emotions. To answer each question in the WOMET, a 100-milimeter line is provided for patients to mark their responses. The total score can range from 0 to 1600, or is expressed as a percentage:

$$ \frac{1600- perso{n}^{\prime }s\  score}{1600}\times 100. $$

Based on percentage scoring, 0 indicates lower quality of life. This instrument was shown to have high reliability (ICC > 0.8) [11].

Because most meniscal ruptures occur during the third, fourth and fifth decades, when people are active, productive and play important social and economic roles in society, due attention is needed to the impact of meniscal injuries, post-traumatic events and rehabilitation on individuals’ quality of life and their social and economic activities. Current methods used to assess meniscal injuries, except for questionnaires, examine only the presence or absence of meniscal pathology, the extent and severity of injury, and the location of the injury. Questionnaires are the only instruments that assess the quality of life and the influence of injuries on individuals’ lives and activities. It is thus important to have a valid and reliable tool that assesses the quality of life of patients after meniscal rupture; moreover, these instruments can determine the effectiveness of different therapiesused to treat meniscal rupture, such as physiotherapy, reconstructive surgery and meniscal resection. The importance of evaluation in identifying patients’ problems and evaluating treatment outcomes makes it necessary to evaluate the validity and reliability of the WOMET, which is the only questionnaire designed to assess quality of life in patients with meniscal pathology. This instrument was found to be better suited to determining quality of life in patients with meniscal rupture than other questionnaires [8]. In the Persian language there is no specific and standard toolto evaluatequality of life in patients with meniscal pathology; hence the aims of the present study were to translation and culturally adaptat the WOMET to Persian, and to test its reliability and validate it for Iranian patients with isolated meniscal injury. Verification of the validity and reliability of the Persian version of WOMET (PWOMET) will make it useful as a specific, standard tool for future research in the field of meniscus damage in the Persian-speaking population.

## Methods

In this cross-sectional test development study, participants were recruited by simple sampling. Sufficient sample size was determined according to the criteria proposed by Terwee et al., i.e. at least 100 patients with isolated meniscal injury and 50 healthy individuals [17]. Healthy individuals in this study were selected from the available population and included after their health status was verified. Patients with isolated meniscal rupture who were eligible for the study and were willing to participate were recruited from hospitals, physiotherapy clinics, clubs and sports centers in Tehran and Shiraz, Iran. Magnetic resonance imaging for each individual were examined by an orthopedist to verify the presence of rupture as an inclusion criterion. Individuals were asked to sign a consent form to participate in the study. The Ethics Committee of Tehran University of Medical Sciences approved the protocol of this research.

The PWOMET, Short Form Health Survey (SF-36), International Knee Documentation Committee (IKDC), visual analogue scale, and Knee Injury and Osteoarthritis Outcome Score (KOOS) instruments were completed by all participants. The SF-36 is a generic qustionnaire for quality of life consisting of 36 items, 8 subscales and 2 components, and is a reliable and valid tool in Iran [18]. The Persian version of the IKDC scale comprises 3 subscales, and has been shown to have good reliability and validity [19]. The KOOS questionnaire was previously translated into Persian [20], and the validity and reliability of this questionnaire for meniscal pathology was reported to be acceptable [21].

Scores on the PWOMET for quality of life were reported according to the percentage for each subscale:

$$ \frac{maximum\ possible\ score\ of\ subscale- perso{n}^{\prime }s\  score}{maximum\ possible\ score\ of\ subscale}\times 100. $$

Demographic information was also recorded by interviewing individuals and entering the data on a demographic data form prepared for this study.

The inclusion criteria were age 18 years or older, isolated meniscal rupture, confirmation of meniscal rupture by magnetic resonance images, ability to read and write Persian, and absence of other knee injuries or problems.

The exclusion criteria were refusal to participate in the research, and new damage to the meniscus during the test-retest interval.

The study was conducted in two stages: the first stage was translation and preparation of the PWOMET, and the second stage was assessment of the validity and reliability of the Persian translation. Translation and adaptation were conducted according to American Association of Orthopedic Surgeons Outcomes Committee guidelines [22]. For the second step we used the checklist in the Consensus-Based Standards for the Selection of Health Status Measurement Instrument (COSMIN) as a guide to evaluating the quality of studies that measure the properties of health-related quality of life assessment instruments [23, 24].

### Stage 1

#### Forward translation

The original English version of the questionnaire was translated into the target language (Persian) by two translators who were native speakers of the target language and had sufficient familiarity with and proficiency in the source language (English). The two translators did not know each other and did not contact each other while they worked on the forward translation.

#### Synthesis

The translators were introduced to each other in a meeting with the lead researchers, and the results of each translation were reviewed to reach a consensus on the initial target language translation.

#### Backward translation

The single translation obtained in the previous step was presented to two other translators who were bilingual and had sufficient fluency in both languages ​​to translate from Persian into English. These two translators were unrelated to each other and were not familiar with the questionnaire.

The two back-translations were combined into a single translation in a meeting attended by the lead researchers. Then the back-translation was compared with the original version of the instrument to ensure that the Persian translation did not differ significantly from the original version. This meeting determined that the Persian version of the questionnaire correctly transmitted all the concepts of the original questionnaire to the patients. The back-translated version was sent to the corresponding author of the original version of the instrument, and was approved by the author.

#### Field test

The final Persian version was tested in a selected sample group of 30 patients with isolated meniscal rupture, and was reviewed by experts who were asked to evaluate its psychological suitability, the ordering and grouping of items, clarity of the meaningof all items and answers, the presence of potentially uncomfortable items, the duration of the questionnaire, and compliance with Iranian culture. After review of the feedback from patients and experts, potential issues in the Persian version were identified and corrected. For example, if an item was flagged as meaningless this item was rewritten to ensure it was comprehensible to participants. If an item or its response options were incompatible with Iranian culture, they were revised to ensure appropriate cultural adaptation. The patients who participated in this stage were referred by orthopedists to physiotherapy clinics in Tehran and Shiraz for rehabilitation for meniscal pathology, and were invited by the researchers to participate in this stage of the study. They were different from the patients who participated in the validity and reliability stage of the present study (see below).

### Stage 2: validity and reliability

The COSMIN checklist contains items that cover 1) validity (content validity, construct validity and criterion validity), 2) reliability (internal consistency, reliability and measurement error), 3) responsiveness, and 4) interpretability [23].

#### Content validity

Content validity is an inidicator of how well the productive items of a questionnaire reflect the intended concept [17]. Content validity of the original version of the WOMET was confirmed by Kirkley et.al [11]. We used a forward translation-back translation-review protocol to produce the PWOMENT and thus assumed that content validity had been preserved, and so did not determine the Content Validity Index, but in accordance with the COSMIN checklist we verified face validity [23, 24] in qualitative form based on the judgment of patients and experts, and in quantitative form based on our estimates of the impact score seperately for each item in the PWOMET. For qualitative face validity, based on the field test results, where more than 15% of people indicated conceptual issues in a particular item, that item was reviewed and rewritten. The impact score was calculated with the formula: Impact score = Frequency (%) × Importance. This formula yields the percentage of participants who indicated that the item was important or quite important on a Likert-like scale. Items that earned an impact score equal to or greater than 1.5 were considered suitable [25, 26].

#### Construct validity

Considering the COSMIN checklist, construct validity can be measured by hypothesis testing and structural validity [23, 24]. We chose 5 hypotheses for our research:

1. We hypothesized that the Physical symptoms subscale of PWOMET would show a moderate positive correlation with the sign subscale of the IKDC, the symptoms subscale of the KOOS, and the physical health component of the SF-36 (convergent validity). Convergent validity indicates the degree of correlation among different measures of the same construct, and is tested with Spearman’s and Pearson’s correlation [27]. For convergent validity, items that presented similar questions and enquired about the same concepts were selected for comparison. For example, the physical symptoms subscale of the WOMET, the sign subscale of the IKDC, the symptoms subscale of the KOOS, and the physical health component of the SF-36 all ask about physical problems and their symptoms. The same rule was applied for other items we examined (hypotheses 1–3).
2. We expected a moderate positive correlation between the Emotions subscale of the PWOMET and the mental health component of the SF-36 (convergent validity).
3. We hypothesized that the Sports/recreation/work/lifestyle subscale of the PWOMET would show a moderate positive correlation with the sport subscale of the IKDC and the recreation subscale of the KOOS (convergent validity).
4. We expected a moderate negative correlation between total score of the PWOMET and a visual analogue scale.
5. We hypothesized that the mean PWOMET score among patients would be lower than the mean score in a healthy group, with a large clinical difference and a mean difference between the two groups that was greater than the standard errorr of measurement (SEM). For this hypothesis the overall score and the scores for each domains of the PWOMET were compared in healthy persons and the meniscal rupture groupwith the independent t-test.

For structural validity we used exploratory factor analysis to extract the structure of the variables.

A Kaiser-Meyer-Olkin test value> 0.6 and Bartlett’s test for sphericity (*p* < 0.05) were considered to indicate sampling adequacy for factor analysis. Any factor with an eigenvalue > 1 was considered significant for factor extraction. The extracted factors were rotated orthogonally with a varimax procedure. Factor loading was considered acceptable at the≥0.40 level [28].

#### Concurrent criterion validity

As explained in the COSMIN, concurrent criterion validity is determined as the relation between a given instrument and a gold standard [17, 24]. A comparison of the PWOMET with a gold standard was desirable, but because there is no gold standard, the correlations between the PWOMET scores and the Persian version of the IKDC, SF-36 and KOOS scores were calculated [17, 24].

#### Reliability

Test-retest reliability indicates the stability of an instrument and its ability to produce similar scores in repeated measurements [17]. Taking into account the COSMIN criteria in the reliability section for the internal consistency of items in the PWOMET, Cronbach’s alpha coefficient was used [17, 23, 24]. Repeatability tests were also performed twice in a 7-day interval in 50 selected patients. To determine the reliability of the instrument, intraclass correlation coefficients (ICC_[2,1]_); (two times and one examiner) were calculated. The acceptable level for ICCs was set at > 0.7 [17].

For measurement errorr, SEM and smallest detectable change were calculated with the formula: $SEM= SD\times \sqrt{1- ICC}$ and $\mathrm{smallest}\ \mathrm{detectable}\ \mathrm{change}=1.96\times SEM\times \sqrt{2\ }$. The SEM shows whether changes in the scoreare real changes or not. For example, changes may be attributable to treatment, pathology, rehabilitation, or measurment error. The smallest detectable change indicates the smallest within-person change in score [17].

#### Interpretability

Interpretability is a standard that can convert a tool’s qualitative score to a quantitative score [17]. This item of the PWOMET was investigated by searching for ceiling and floor effects, and by calculating the minimal important difference with the formula 0.5 × SD based on the COSMIN checklist [24]. To check ceiling or floor effects, if more than 15% of participants had a total score higher than 80% or lower than 20%in the PWOMET, the instrument was considered to have a ceiling or floor effect [17].

Descriptive statistics for continuous variables are presented as the mean, standard deviation, median, minimum and maximum. Nominal variables (such as sex, affected side, type of problem, etc.) were expressed in percentages and absolute numbers. For analytical statistics, the Kolmogrov-Smirnov test was used to check the distribution of quantitative variables. Pearson’s and Spearman’s correlation coefficients were used to determine construct validity and concurrent criterion validity. Correlations lower than 0.40, between 0.40 and 0.70, and greater than 0.70 were considered as weak, moderate and strong, respectively. A *p*-value lower than 0.05 was regareded as statistically significant. SPSS software version 20.0 was used for all statistical analyses.

## Results

Among the 100 patients 29% were female (mean age ± SD: 36.66 ± 10.15) and 71% were male (mean age ± SD: 30.62 ± 9.42). Among the 50 healthy people 68% were female (mean age ± SD: 26.82 ± 8.06) and 32% were male (mean age ± SD: 31.81 ± 9.70). There were no significant differences between groups in demographic data (age, height, weight). The frequencies of patients, mean visual analogue scale scores and total WOMET scores according to the affected side and type of pathology are shown in the Table 1.

**Table 1:** Frequency of patients and mean ± standared deviation of visual analogue scale scores and total scores on the Persian Western Ontario Meniscal Evaluation Tool

	Sex			Type of problem			Affected side	
	Total (*N* = 100)	Female (*N* = 29%)	Male (*N* = 71%)	Medial menisc (*N* = 60%)	Lateral menisc (*N* = 36%)	Both (*N* = 4%)	Right knee (*N* = 60%)	Left knee (*N* = 40%)
TotalPWOMETscore (mean ± SD)	37.39 ± 18.15	39.68 ± 16.07	31.79 ± 21.74	35.29 ± 18.75	40.27 ± 17.61	42.92 ± 15.36	36.74 ± 18.90	38.37 ± 17.15
Visual analogue scale	46.76 ± 29.29	53.14 ± 29.23	44.15 ± 29.12	52.70 ± 29.00	36.17 ± 26.75	53.00 ± 36.19	47.73 ± 29.93	45.30 ± 28.61

*PWOMET* Persian version of Western Ontario Meniscal Evaluation Tool, *SD* Standard deviation

Mean pain scores on the visual analogue scale were 46.76 ± 29.29 in the patient group and 12.32 ± 21.06 in the healthy group.

Table 2 summarizes descriptive statistics for the WOMET subscales in both groups. Minimum and maximum scores for the WOMET are 0 and 100, respectively. Scores nearer to 0 indicate lower quality of life [11].

**Table 2:** Descriptive statistics for the Persian Western Ontario Meniscal Evaluation Tool

Items	Health status	Mean ± SD	Median (range)	95%CI		Health status	Mean ± SD	Median (range)	95%CI	
				Lower bound	Upper bound				Lower bound	Upper bound
**Physical symptoms**	Patients (*N* = 100)	46.33 ± 21.51	45.11 (0.00–100.00)	42.06	50.59	Healthy (*N* = 50)	86.78 ±15.04	90.61 (29.33–100.00)	82.51	91.06
**Sports/recreation/work/lifestyle**	Patients (*N* = 100)	24.87 ± 20.30	20.88 (0.00–83.75)	20.84	28.89	Healthy (*N* = 50)	87.42 ±20.32	98.50 (29.75–100.00)	81.64	93.19
**Emotions**	Patients (*N* = 100)	27.27 ± 20.46	27.17 (0.00–91.67)	23.21	31.33	Healthy (*N* = 50)	71.39 ±23.52	73.17 (20.33–100.00)	64.71	78.08
**Total score of PWOMET**	Patients (*N* = 100)	37.39 ± 18.15	34.50 (0.00–88.69)	33.79	40.99	Healthy (*N* = 50)	84.06 ±16.24	89.47 (30.44–100.00)	79.44	88.67

*PWOMET* Persian version of Western Ontario Meniscal Evaluation Tool, *CI* Confidence interval, *SD* Standard deviation

### Content validity

After review of the PWOMET, 20% of the evaluators indicated that the item “How conscious are you of your knee?” should be rewritten. The impact score of this and all other items was greater than 1.5 points. This item was changed to “How much attention do you give to your knee?”. In the responses to this item, the “extremely conscious” item was changed to “a great deal of attention”. In the instructions, the sentence “Please indicate your answer with a slash across the horizontal line” was replaced with “Please indicate your answer for each question with a slash on the line”.

### Construct validity

We developed 5 hypotheses for construct validity, and according to the data summarized in Table 3 and other findings, all hypotheses were supported.

**Table 3:** Correlation between the Persian Western Ontario Meniscal Evaluation Tool and related subscales of other questionnaires (*N* = 100)

	IKCD		SF-36		KOOS	
	Sign	Sport	Physical health component	Mental health component	Symptoms	Recreation
**Physical symptoms**	0.52 (0.0001)^a^		0.42 (0.0001)^a^		0.57 (0.0001)^b^	
**Sports / recreation / work / lifestyle**		0.51 (0.0001)^a^				0.58 (0.0001)^a^
**Emotions**				0.46(0.0001)^a^		

Correlations were significant at the 0.01 level (two-tailed)

*PWOMET* Persian versin of Western Ontario Meniscal Evaluation Tool, *SF-36* Short Form Health Survey, *IKDC* International Knee Documentation Committee, *KOOS* Knee Injury and Osteoarthritis Outcome Score

^a^Spearman correlation; ^b^ Pearson correlation

The correlations between WOMET subscale scores and scores on the SF-36, IKDC and KOOS subscales are shown in Table 3.

Scores on the visual analogue scale showed a moderate negative correlation with the PWOMET (*r* = − 0.47, *p* < 0.001). The independent t-test was used to search for differences in the PWOMET score between the healthy and patient groups. This analysis showed significant differences between groups (t = − 15.36, *p* < 0.0001, mean difference = − 46.67, 95% confidence interval: − 52.67 to− 40.66). The scores on all three PWOMET subscales also differed significantly (*p* < 0.0001).

**Fig. 1:**
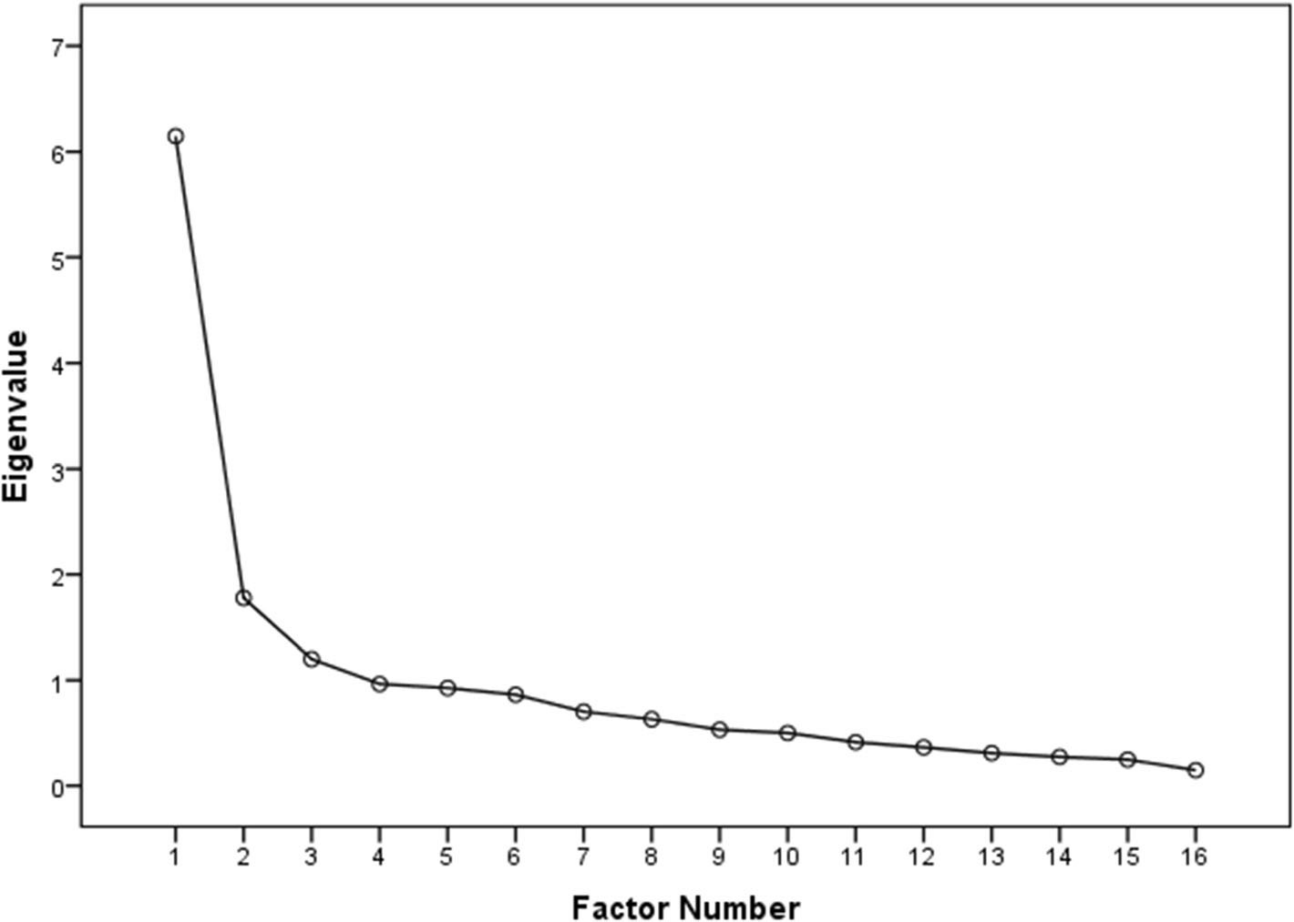
Scree plot for the Persian version of the WOMET

Structural validity was evaluated by exploratory factor analysis in the 100 participants with meniscal pathology. The Kaiser-Meyer-Olkin test and Bartlett’s test demonstrated that the data were appropriate and sample size was adequate for factor analysis (Kaiser-Meyer-Olkin index = 0.81, χ2 = 696.010, *p* < 0.0001). Factor analysis with varimax rotation identified 3 factors with eigenvalues greater than 1 and factor loading equal to or greater than 0.5, accounting for 57.03% of the variance observed. The factor loadings were categorized as factor 1including 7 items (items 1, 2, 3, 4, 6, 7 and 8), factor 2 including 6 items (items 10, 11, 12, 13, 14 and 15), and factor 3 including all other items. The scree plot confirmed retention of the first three factors, with eiganvalues > 1 (Fig. 1).

### Concurrent criterion validity

Pearson’sand Spearman’scorrelation were used for concurrent criterion validity. The correlations between PWOMET total score andscores on the SF-36 and KOOS were 0.54 (*p* < 0.001) and 0.63 (*p* < 0.001), respectively. Spearman’s correlation for the PWOMET and the IKDC yielded a coefficient of 0.61 (*p* < 0.001).

### Reliability

Fifty patients completed PWOMET a second time after 1 week to provide data for reliability. They did not start any treatment and were not diagnosed with any new impairment during this week. Table 4 provides descriptive statistics for PWOMET scores in the second assessment. The paired t-test used to investigate test-retest differences disclosed no significant difference between the two sets of scores (*p* = 0.41, mean difference = − 1.68, 95%CI: − 5.76 to 2.40). Pearson’scorrelation coefficient for the first and second measurements was calculated as *r* = 0.73 (*p* < 0.0001).

**Table 4:** Descriptive statistics for the Persian Western Ontario Meniscal Evaluation Tool (retest, *N* = 50)

	Mean ± SD	Median (range)	95%CI	
			Upper bound	Lower bound
**Physical symptoms**	47.47 ± 23.18	42.44 (8.44–95.67)	54.06	40.89
**sports / recreation / work / lifestyle**	32.22 ± 21.96	25.50 (6.00–81.75)	38.47	25.98
**Emotions**	34.56 ± 20.46	32.67 (0.00–90.33)	40.37	28.75
**Total score onPWOMET**	41.24 ± 19.89	35.66 (7.69–85.56)	46.89	35.59

*SD* Standard deviation, *CI* Confidence interval, *PWOMET* Persian version of Western Ontario Meniscal Evaluation Tool

The SEM for total PWOMET score was 9.43; for each subscale SEM was Physical symptoms 10.31, Sports/recreation/work/lifestyle 12.34, and Emotions 10.23 (Table 5).

**Table 5:** Intraclass correlation coefficient, standard error of measurement, smallest detectable change for Persian Western Ontario Meniscal Evaluation Tool (retest, *N* = 50)

	ICC(95%CI)	SEM^a^	Smallest detectable change
**Physical symptoms**	0.77 (0.63–0.86)	10.31	28.57
**Sports / recreation / work / lifestyle**	0.63 (0.43–0.77)	12.34	34.20
**Emotions**	0.75 (0.60–0.85)	10.23	28.35
**Total score onPWOMET**	0.73 (0.56–0.83)	9.43	26.13

*CI* Confidence interval, *ICC* Intraclass correlation coefficient, *SEM* Standard error of measurement, *PWOMET* Persian version of Western Ontario Meniscal Evaluation Tool

^a^SD used to calculate SEM is related to 100 participants

The overall Cronbach’s alpha coefficient for the PWOMET was 0.89; for each subscale this coefficient was Physical symptoms 0.85, Sports/recreation/work/lifestyle 0.77, and Emotions0.61. Spearman’scorrelation between total PWOMET score and its subscale scores was r = 0.50 to 0.93 (*p* < 0.0001). Individual PWOMET item scores showed significant correlations with the total score (*r* = − 0.46 to− 0.77, *p* < 0.001).

### Interpretability

In the PWOMET, 3 persons scored> 80% and 14 persons scored< 20%, indicating the absence of any ceiling or floor effect. In the healthy group, 76% of participants scored higher than 80% of the maximum possible score. Minimal important difference for the PWOMET total scores was 9.07 points; for each subscale this parameter was Physical symptoms 10.75, Sport/recreation/work/lifestyle 10.15, and Emotions 10.23 points.

## Discussion

Instruments to evaluate health status have been developed mainly for use in English-speaking countries. It is important to properly translate, culturally adapt and evaluate non-English-language instruments such as questionnaires in order to compare the results of health status assessments in different cultural groups, and the results of trials in different countries [29–31]. Until now, no validated disease-specific measure was available to assess quality of life in Persian-speaking patients with meniscal pathology. The WOMET is a self-administered instrument that is easy to complete, and the present study investigated the results of the into-Persian translation and cultural adaptation of theWOMET. The translation and validation procedures presented no problems, and the resulting PWOMET showed good content retention, and good reliability and validity scores. Persian-speaking investigators and clinicians can now use this version of the test with confidence.

### Content validity

All participants responded to all items, and there were no missing data. As found previously for the English [11], Turkish [12], Finnish [13], German [15] and Chinese [14] versions, acceptability and understandability of the PWOMET were confirmed in our sample of patients with meniscal pathology.

### Construct validity

Construct validity ofthe PWOMET was acceptable. Mean score was significantly lower in our patient group than the healthy group, indicating that patients with meniscal pathology had a lower quality of life. This finding is consistent with results reported for the Finnish version [13]. The mean differences in total and subscale scores on the PWOMET were shown within dependent t-tests to be larger than the minimal important difference and the SEM, indicating that the differences between our healthy and patient groups were not random and were clinically significant.

Structural validity of the PWOMET was assessed with exploratory factor analysis. Our finding of 3 components parallels the structure reported for the original version of the WOMET [11], with only small differences in factor 3. Items 5 and 9 are in factor 1 in the original version of the questionnaire, but these two items loaded on factor 3 in the present study. Items 14 and 15, which are part of factor 3 in the original version, loaded on factor 2 in our study. These differences may be related to differences between Iranian and English cultures. This component of the PWOMET can be designated with a new label, e.g. “Knee problems and depression”. None of the reports on other language versions of the WOMETinvestigated structural validity, so we were unable to further compare our results with those of earlier publications.

### Concurrent criterion validity

The correlation between thePWOMET and the SF-36, KOOS, IKDC were moderate to good. These significant, acceptable correlations confirm the concurrent criterion validity of the PWOMET. It should be noted that SF-36 is a generic questionnaire, whereas the WOMET is specific test, so it was not surprising that the relationship between these two instruments was not as strong as for the KOOS or IKDC. These results are consistent with other versions of the test, and the few differences seen across versions may be due to differences in cultures, beliefs, and living conditions of populations with different languages. The correlation for concurrent criterion validity of other language versions of the WOMET was 0.11–0.68 for the SF-36, 0.41–0.72 for the KOOS, and 0.68–0.76 for the IKDC [12, 14–16].

### Reliability

The correlation between scores in the first and second trials showed that the PWOMET has acceptable test-retest reliability. As in other language versions of the WOMET, the intraclass correlation of the PWOMET (0.73) was also good. The ICC for other language versions was 0.85 for theEnglish version, 0.86 for Turkish, 0.90 for German, 0.78 for Dutch, and 0.93 for Chinese [11, 12, 14–16]. The ICC for the Sports/recreation/work/lifestyle item in the PWOMET was 0.63, very similar to the 0.65 correlation found for the Dutch version [16]. Repetition and averaging of scores of this subscale, the use of average ICC, and adding parallel questions to the Sports/recreation/work/lifestyle subscale can cover this weakness. Changing the scoring system for the WOMET (for example, by using a numerical or qualitative rating scale rather than a visual analogue scale) may affect its psychometric properties and ICC. The smallest detectable change we obtained for the PWOMET means that a 26.13-point change in the total PWOMET score indicates that the treatment or intervention was clinically meaningful.

Cronbach’s alpha coefficient for the PWOMET total and subscale scores was greater than 0.7, thus demonstrating a high correlation among items, and supporting the internal consistency of this tool. Other language versions of the WOMET also reported an internal consistency above 0.7, e.g. English 0.92, Turkish 0.89, Finnish 0.91, German 0.92, and Chinese 0.9 [11–15]. The correlation between total WOMET score and its subscale scores was moderate to high, indicating good stability of all items.

### Interpretability

The absenceof ceiling or floor effects confirmed good interpretability and content validity of the PWOMET. The other versions of the WOMET likewise had no ceiling or floor effects (0 to 5.7%) for total score [11–16]. In the healthy group, the ceiling effectwas reasonable and predictable because these participants presumably have a good quality of life, so their responses would be expected to yield high scores.

The Persian KOOS has shown good reliability and validity for meniscal injuries [21], but although the reliability and validity of the KOOS and other instruments are acceptable, it should be noted that the WOMET has more items which are more specific for people with meniscal pathology than other assessment tools, e.g., items about knee awareness and attention, numbness in and around the knee, and pain after weight bearing. Moreover, the WOMET requires a shorter time to complete than the KOOS, which consists of 42 items and is a time-consuming tool related to knee problems and osteoarthritis generally. The PWOMET meets the need in Persian-speaking populations for adedicated tool for meniscal injury assessment that can be completed in a short time.

A tool to assess quality of life in pathologic conditions is essential to evaluatethe effectivness of treatments before and after intervention. Many disease-specific tools are available for health-related quality of life and functional status in patients with meniscal pathology, but their measurement properties are often generic rather than specific. Both generic and disease-specific patient-reported outcome measurements can be used for patients with isolated meniscal pathology, but the latter are often considered more sensitive than generic patient-reported outcome measurements, because they are developed specifically for well-defined patient populations. The WOMET is the first tool to specifically assess health-related quality of life in patients with meniscal injury. This instrument is better able to detect meniscal tears and measure their effect on quality of life than other instruments. In addition, the WOMET score, unlike other instruments, does not showgender-related differences [8]. The WOMET has the highest content validity among instruments used to assess meniscal injury problems [9]. A further advantage is that it consists of fewer but more informative items, and is consequently faster to complete for patients. The analysis reported here shows that the PWOMET has good reliability and validity, and can thus be used by Persian investigators. The PWOMET fills the need for a specific standard tool for meniscal injury in Persian-speaking patients. When faced with the need to compare different available interventions for meniscus injuries, researchers, orthopedists, and other clinicians in Persian-speaking settings can use the PWOMET to evaluate the outcomes, cost effectiveness and impact of different treatment methods on patients and their quality of life. This instrument can be used in two ways. 1) Total PWOMET score is useful as a single index score to assess quality of life related to meniscal injury, and the trend in quality of life. 2) Subscale scores can provide more specific and detailed information about quality of life in patients with meniscal injury, in the areas of Physical symptoms, Sports/recreation/work/lifestyle, and Emotions.

The main limitation of the present study is the lack of a specific standard in Persian for comparison with the WOMET. In addition, we did not classify our population sample based on the grade of meniscal pathology. All patients were in the preoperative stage, so the results may differ for patients in the postoperative stage and among those treatedwith different types of surgery. Further studies are advisable to investigate other properties of the WOMET such as responsiveness.

## Conclusion

The WOMET is the only instrument designed specifically for meniscal injuries. Based on the results reported here, the Persian version of the WOMET provides valid and reliable scores for the evaluation of quality of life in patients with meniscal pathology as a single index score and also in three specific areas. Researchers, physical therapists, orthopedists and surgeons can use this validatedtool as a specific standard in their research and in the clinical assessment of the results of interventions provided to individuals with meniscal injuries.

## Data Availability

The data file of this study is available from the corresponding author and will be made available to anyone upon reasonable request.

## Abbreviations

COSMIN: Consensus-Based Standards for the Selection of Health Status Measurement Instrument
ICC: Intraclass correlation coefficient
IKDC: International Knee Documentation Committee
KOOS: Knee Injury and Osteoarthritis Outcome Score
PWOMET: Persian version of Western Ontario Meniscal Evaluation Tool
SEM: Standard error of measurement
SF-36: Short Form Health Survey
WOMET: Western Ontario Meniscal Evaluation Tool
